# Longitudinal trajectories of metabolic syndrome on different neurocognitive domains: a cohort study from the Taiwan biobank

**DOI:** 10.18632/aging.203099

**Published:** 2021-06-11

**Authors:** Shou-En Wu, Wei-Liang Chen

**Affiliations:** 1 Division of Family Medicine, Department of Family and Community Medicine, Tri-Service General Hospital, School of Medicine, National Defense Medical Center, Taipei, Taiwan, Republic of China; 2 Division of Geriatric Medicine, Department of Family and Community Medicine, Tri-Service General Hospital, School of Medicine, National Defense Medical Center, Taipei, Taiwan, Republic of China; 3 Department of Biochemistry, National Defense Medical Center, Taiwan, Republic of China

**Keywords:** metabolic syndrome, mild cognitive impairment, dementia, Taiwan biobank

## Abstract

Metabolic syndrome (MetS) brings considerable effects on cognitive function, but trajectories within remain unclear. We investigated the interactions between distinct MetS components and cognitive domains. A total of 5693 participants from the Taiwan biobank during 2008–2018 were enrolled. Participants were classified as either normal or as having MetS at two time points; i.e., study entry and follow-up. At both the time points, cognitive evaluations using the Mini-Mental State Examination (MMSE) were conducted. The hazard ratios (HRs) of mild cognitive impairment (MCI) and dementia were higher in participants meeting more diagnostic components of MetS. Of the five criteria of MetS, three were significantly associated with MCI and dementia: high blood pressure (MCI: HR = 1.203, *p* < 0.001; dementia: HR = 1.345, *p* < 0.001), abdominal obesity (MCI: HR = 1.137, *p* = 0.006; dementia: HR = 1.442, *p* < 0.001), and low high-density lipoprotein (HDL) level (MCI: HR = 1.149, *p* = 0.007; dementia: HR = 1.364, *p* < 0.001). Of the cognitive domains measured, three were significantly associated with MetS; namely, orientation, language, and visuospatial abilities. Participants who were initially diagnosed with MetS but were normal at follow-up had an HR of 1.374 for dementia (*p* = 0.019), which was beyond our expectations. The undiminished risk of cognitive decline in subjects returning to normal status illustrated that neural injury caused by MetS takes a long time to get repaired. Consequently, earlier detection and management of adjustable risk factors of MetS should be encouraged to minimize the damage.

## INTRODUCTION

Metabolic syndrome (MetS) is a complex disorder characterized by a cluster of cardiometabolic dysfunctions including impaired glucose tolerance, dyslipidemia, hypertension, and central obesity [1]. With the number of cases increasing globally, MetS is now considered a global pandemic [2]. While atherosclerotic cardiovascular disease and type 2 diabetes mellitus (DM) are known consequences of MetS, its role in numerous diseases has been verified [3–5]. Among them, the relationship between MetS and cognitive decline is an important area of ongoing research.

A 5-year prospective observational study involving 2632 community-dwelling elderly people in the US determined that MetS contributes to cognitive impairment by significant high level of inflammation [6]. Bokura et al described the association between MetS and impaired executive function independent of silent brain lesions [7]. Plentiful studies have highlighted this issue, which has been summarized by several review articles [8, 9]. Proposed underlying mechanisms linking MetS and cognitive impairment include chronic inflammation [10], excessive reactive oxygen species (ROS) [11], vascular endothelial damage [12], and insulin resistance [13]. However, the interactions between separate MetS components and cognitive domains are not much studied. Some previous studies have tried to demonstrate the impact of individual metabolic index on cognition, but most of these studies only revealed the risk by viewing MetS as a whole or in less than two indices [14–17]. On the other hand, most studies focused on the risk accompanying MetS [14, 15]; however, few studies investigated if the restoration of MetS to normal status could alter the risks. The present study investigated the inter-correlations of detailed elements (including both MetS indices and cognitive domains) and the effects of transitioning from normal to MetS and vice versa on cognitive domains. Our findings provide insight into the fluctuation of risk of developing cognitive deficits based on metabolic function.

## RESULTS

### Lower MMSE scores are presented in participants with MetS

Data from the Taiwan biobank showed that MMSE scores were lower in the MetS group than in the normal group both at baseline (26.96 ± 2.68, *p* = 0.003) and at follow-up (27.26 ± 2.51, *p* < 0.001) (Table 1).

**Table 1:** Demographic information of participants with and without metabolic syndrome at baseline.

	**MetS (*n* = 1565)**	**Normal (*n* = 4128)**	***p* value**
Continuous variables			
Age	63.74 ± 2.78	63.60 ± 2.73	0.077
Waist circumference	90.96 ± 8.39	83.17 ± 8.53	<0.001
Body fat percentage	31.87 ± 7.13	27.20 ± 7.40	<0.001
BMI	26.23 ± 3.20	23.57 ± 2.93	<0.001
SBP	138.84 ± 18.61	124.77 ± 17.75	<0.001
DBP	79.03 ± 1.42	72.96 ± 10.45	<0.001
HDL	45.16 ± 9.94	56.88 ± 3.11	<0.001
TG	175.53 ± 104.89	97.41 ± 46.88	<0.001
Fasting glucose	112.82 ± 32.21	96.83 ± 15.78	<0.001
MMSE (baseline)	26.96 ± 2.68	27.19 ± 2.58	0.003
Categorical variables			
male	36.6%	41.2%	0.002
Education (>6 years)	78.6%	83.5%	<0.001
CAD	15.9%	17.0%	0.291
Dyslipidemia	17.0%	15.9%	<0.001
HTN	69.3%	58.9%	<0.001
DM	51.2%	38.3%	<0.001
Smoking history	23.8%	22.9%	0.494

Abbreviations: BMI: Body mass index; SBP: Systolic blood pressure; DBP: Diastolic blood pressure; HDL: High-density lipoprotein cholesterol; TG: Triglyceride; MMSE: Mini-Mental State Examination; CAD: Coronary artery disease; HTN: Hypertension; DM: Diabetes mellitus.

### Hazard ratios of MCI and dementia in five diagnostic components of MetS.

**Figure 1:**
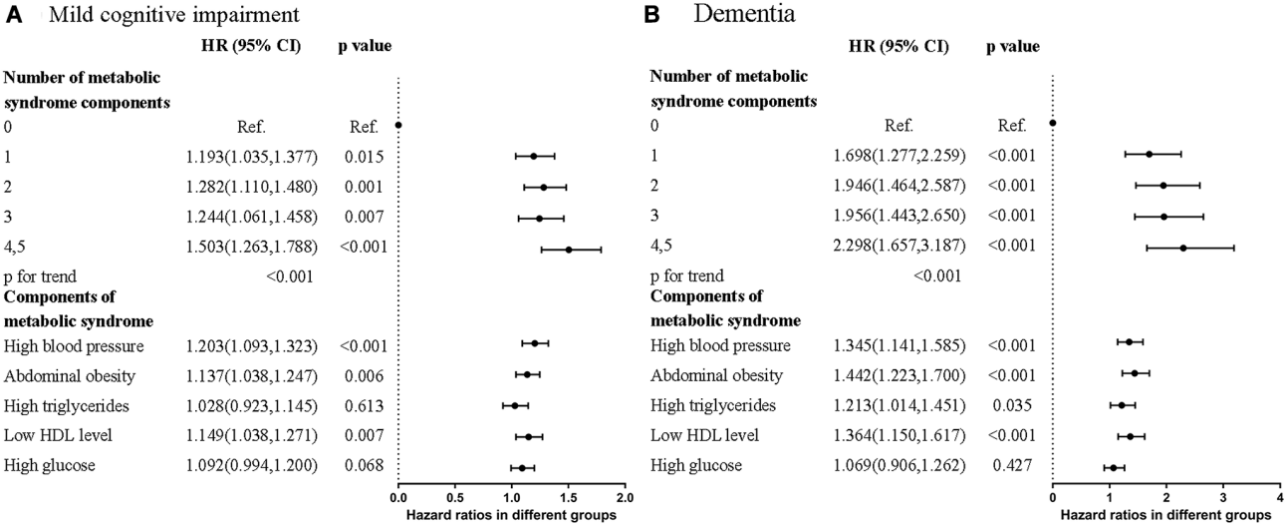
Forest plots that showed hazard ratios of (**A**) Mild cognitive impairment (MCI) (**B**) Dementia in different numbers and contents of metabolic syndrome components.

Hazard ratios (HRs) of MCI and dementia were higher in participants who met more diagnostic components of MetS (Figure 1 and Supplementary Table 1). In participants who met at least four of the MetS criteria, HRs were 1.503 (95% CI = 1.263–1.788, *p* < 0.001) for MCI and 2.298 (95% CI = 1.657–3.187, *p* < 0.001) for dementia compared to the normal group. The HRs for dementia were higher than those for MCI in all subgroups.

**Figure 2:**
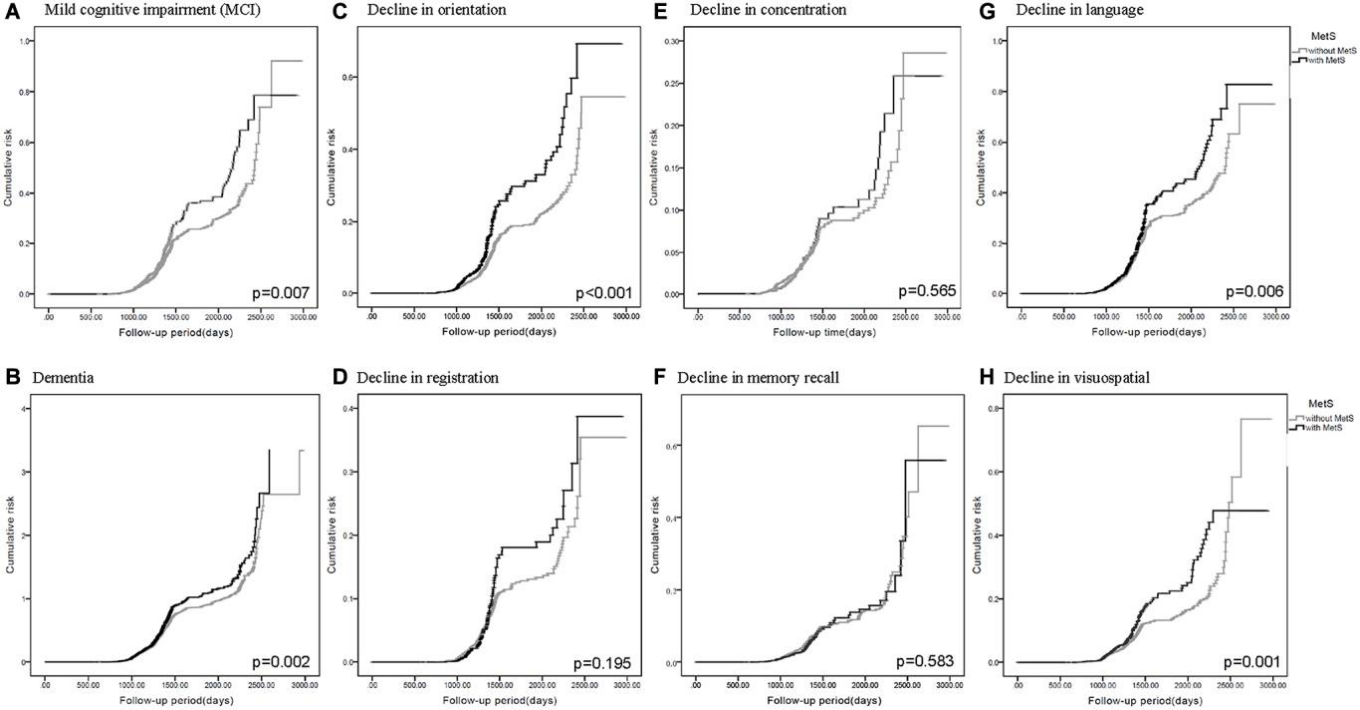
**Kaplan-Meier curves for the cumulative risk of cognitive decline in participants with and without metabolic syndrome (MetS).** Different cognitive evaluations include: (**A**) Mild cognitive impairment (MCI) (*p* = 0.007) (**B**) Dementia (*p* = 0.002) (**C**) Decline in orientation (*p* < 0.001) (**D**) Decline in registration (*p* = 0.195) (**E**) Decline in concentration (*p* = 0.565) (**F**) Decline in memory recall (*p* = 0.583) (**G**) Decline in language (*p* = 0.006) (**H**) Decline in visuospatial (*p* = 0.001).

Of the five diagnostic components of MetS, three were significantly associated with MCI and dementia. They were high blood pressure (MCI: HR = 1.203, 95% CI = 1.093–1.323, *p* < 0.001; dementia: HR = 1.345, 95% CI = 1.141–1.585, *p* < 0.001), abdominal obesity (MCI: HR = 1.137, 95% CI = 1.038–1.247, *p* = 0.006; dementia: HR = 1.442, 95% CI = 1.223–1.700, *p* < 0.001), and low HDL level (MCI: HR = 1.149, 95% CI = 1.038–1.271, *p* = 0.007; dementia: HR = 1.364, 95% CI = 1.150–1.617, *p* < 0.001) compared to the normal group. The Kaplan–Meier curves revealed higher risk of MCI and dementia in participants with MetS (Figure 2A and 2B). Collectively, participants who met more MetS criteria had a higher risk of MCI and dementia, and the three most closely-related components were high blood pressure, abdominal obesity, and low HDL level.

### Hazard ratios for decline in different cognitive domains

HRs predicted lower scores in the orientation, language, and visuospatial abilities domains of the MMSE in participants with MetS (Tables 2 and 3). In the same manner as mentioned previously, HRs were higher in groups possessing more diagnostic components of MetS (Table 2), and revealed significance in the three closely-related metabolic components (Table 3). In addition, the Kaplan–Meier curves revealed higher cumulative risk of decline in orientation (Figure 2C), language (Figure 2G), and visuospatial abilities (Figure 2H) in participants with MetS. Other domains in MMSE including registration (Figure 2D), concentration (Figure 2E), and memory recall (Figure 2F) didn’t revealed statistical significance.

**Table 2:** Hazard ratios of the presence and number of metabolic syndrome(MetS) components for decline in different cognitive domains.

**Variables**		**Orientation**		**Registration**		**Concentration**		**Memory recall**		**Language**		**Visuospatial abilities**	
		**HR (95% CI)**	***p* value**	**HR (95% CI)**	***p* value**	**HR (95% CI)**	***p* value**	**HR (95% CI)**	***p* value**	**HR (95% CI)**	***p* value**	**HR (95% CI)**	***p* value**
Model 1	No MetS	Ref.	Ref.	Ref.	Ref.	Ref.	Ref.	Ref.	Ref.	Ref.	Ref.	Ref.	Ref.
	Presence of MetS	1.577 (1.303,1.909)	<0.001	1.187 (0.920,1.529)	0.187	1.080 (0.808,1.444)	0.601	0.919 (0.703,1.203)	0.541	1.240 (1.058,1.454)	0.008	1.411 (1.141,1.744)	0.001
	Number of metabolic syndrome components												
	1	1.194 (0.879,1.621)	0.257	1.275 (0.880,1.846)	0.199	2.303 (1.418,3.740)	0.001	1.575 (1.068,2.321)	0.022	1.238 (0.970,1.579)	0.086	1.340 (0.950,1.891)	0.096
	2	1.222 (0.895,1.668)	0.208	1.203 (0.820,1.763)	0.345	2.283 (1.397,3.731)	0.001	1.790 (1.215,2.637)	0.003	1.414 (1.109,1.804)	0.005	1.569 (1.114,2.209)	0.010
	3	1.704 (1.241,2.339)	0.001	1.296 (0.860,1.954)	0.215	2.441 (1.456,4.091)	0.001	1.390 (0.896,2.155)	0.141	1.434 (1.103,1.864)	0.007	1.613 (1.117,2.329)	0.011
	4,5	2.019 (1.434,2.843)	<0.001	1.576 (1.009,2.462)	0.045	1.589 (0.846,2.984)	0.150	1.374 (0.829,2.279)	0.218	1.713 (1.289,2.275)	<0.001	2.343 (1.601,3.428)	<0.001
	*p* for trend	<0.001		0.080		0.113		0.326		<0.001		<0.001	
Model 2	No MetS	Ref.	Ref.	Ref.	Ref.	Ref.	Ref.	Ref.	Ref.	Ref.	Ref.	Ref.	Ref.
	Presence of MetS	1.455 (1.201,1.762)	<0.001	1.154 (0.895,1.489)	0.269	0.959 (0.717,1.282)	0.777	0.875 (0.669,1.146)	0.333	1.165 (0.994,1.367)	0.060	1.280 (1.034,1.583)	0.023
	Number of metabolic syndrome components												
	1	1.106 (0.814,1.502)	0.519	1.259 (0.869,1.824)	0.223	2.060 (1.268,3.346)	0.004	1.524 (1.033,2.247)	0.034	1.172 (0.918,1.495)	0.203	1.211 (0.858,1.709)	0.277
	2	1.043 (0.762,1.427)	0.792	1.150 (0.783,1.688)	0.477	1.802 (1.099,2.956)	0.020	1.699 (1.151,2.506)	0.008	1.252 (0.980,1.599)	0.072	1.316 (0.932,1.858)	0.118
	3	1.455 (1.058,2.001)	0.021	1.235 (0.818,1.864)	0.315	1.935 (1.151,3.252)	0.013	1.280 (0.825,1.987)	0.271	1.273 (0.978,1.657)	0.072	1.338 (0.925,1.937)	0.122
	4,5	1.678 (1.190,2.368)	0.003	1.504 (0.961,2.352)	0.074	1.207 (0.641,2.272)	0.561	1.277 (0.769,2.120)	0.345	1.488 (1.118,1.979)	0.006	1.875 (1.279,2.750)	0.001
	*p* for trend	<0.001		0.144		0.758		0.586		0.005		0.002	
Model 3	No MetS	Ref.	Ref.	Ref.	Ref.	Ref.	Ref.	Ref.	Ref.	Ref.	Ref.	Ref.	Ref.
	Presence of MetS	1.447 (1.192,1.756)	<0.001	1.146 (0.886,1.482)	0.298	0.942 (0.703,1.263)	0.691	0.853 (0.650,1.120)	0.251	1.170 (0.995,1.374)	0.057	1.268 (1.022,1.573)	0.031
	Number of metabolic syndrome components												
	1	1.113 (0.819,1.513)	0.492	1.254 (0.865,1.818)	0.232	2.058 (1.267,3.345)	0.004	1.514 (1.026,2.233)	0.037	1.180 (0.924,1.506)	0.184	1.212 (0.859,1.712)	0.274
	2	1.048 (0.765,1.436)	0.769	1.145 (0.778,1.684)	0.493	1.782 (1.085,2.929)	0.023	1.662 (1.124,2.458)	0.011	1.265 (0.989,1.617)	0.061	1.311 (0.927,1.853)	0.126
	3	1.446 (1.049,1.994)	0.024	1.224 (0.808,1.856)	0.340	1.899 (1.126,3.203)	0.016	1.236 (0.793,1.927)	0.349	1.288 (0.988,1.681)	0.062	1.327 (0.914,1.927)	0.136
	4,5	1.692 (1.195,2.396)	0.003	1.491 (0.949,2.343)	0.083	1.180 (0.625,2.230)	0.610	1.244 (0.746,2.075)	0.403	1.509 (1.131,2.014)	0.005	1.867 (1.268,2.749)	0.002
	*p* for trend	0.001		0.163		0.860		0.731		0.005		0.002	

Model 1 = unadjusted.

Model 2 = Model 1 + age, gender, and education level.

Model 3 = Model 2 + (smoking, coronary artery disease, hypertension, type 2 DM).

Abbreviations: HR: Hazard ratio; MetS: Metabolic syndrome.

**Table 3:** Hazard ratios of components of metabolic syndrome for decline in different cognitive domains.

**Variables**		**Orientation**		**Registration**		**Concentration**		**Memory recall**		**Language**		**Visuospatial abilities**	
		**HR (95% CI)**	***p* value**	**HR (95% CI)**	***p* value**	**HR (95% CI)**	***p* value**	**HR (95% CI)**	***p* value**	**HR (95% CI)**	***p* value**	**HR (95% CI)**	***p* value**
Model 1	No MetS	Ref.	Ref.	Ref.	Ref.	Ref.	Ref.	Ref.	Ref.	Ref.	Ref.	Ref.	Ref.
	High blood pressure	1.349 (1.115,1.631)	0.002	1.101 (0.859,1.411)	0.448	0.993 (0.749,1.316)	0.959	1.247 (0.977,1.591)	0.076	1.310 (1.124,1.526)	0.001	1.271 (1.031,1.567)	0.025
	^*^Abdominal obesity	1.335 (1.105,1.613)	0.003	1.217 (0.960,1.545)	0.105	1.561 (1.187,2.053)	0.001	0.984 (0.779,1.243)	0.890	1.244 (1.070,1.447)	0.004	1.396 (1.134,1.718)	0.002
	High triglycerides	1.128 (0.914,1.393)	0.262	0.928 (0.700,1.230)	0.602	1.245 (0.927,1.670)	0.145	0.947 (0.716,1.253)	0.704	0.992 (0.833,1.182)	0.928	1.140 (0.906,1.435)	0.264
	Low HDL level	1.516 (1.249,1.841)	<0.001	1.235 (0.957,1.594)	0.105	1.061 (0.790,1.425)	0.694	1.031 (0.790,1.346)	0.822	1.378 (1.177,1.613)	<0.001	1.432 (1.156,1.775)	0.001
	High glucose	1.186 (0.982,1.432)	0.077	1.147 (0.901,1.459)	0.266	0.839 (0.675,1.182)	0.429	1.256 (0.990,1.594)	0.060	1.120 (0.961,1.305)	0.147	1.320 (1.077,1.619)	0.008
Model 2	No MetS	Ref.	Ref.	Ref.	Ref.	Ref.	Ref.	Ref.	Ref.	Ref.	Ref.	Ref.	Ref.
	High blood pressure	1.325 (1.095,1.605)	0.004	1.041 (0.811,1.335)	0.755	0.946 (0.713,1.255)	0.699	1.177 (0.922,1.503)	0.192	1.282 (1.100,1.495)	0.002	1.257 (1.018,1.551)	0.033
	^*^Abdominal obesity	1.090 (0.895,1.327)	0.392	1.278 (0.998,1.637)	0.052	1.181 (0.887,1.573)	0.254	1.014 (0.796,1.293)	0.909	1.079 (0.922,1.263)	0.342	1.093 (0.881,1.356)	0.419
	High triglycerides	1.083 (0.876,1.338)	0.462	0.901 (0.679,1.196)	0.471	1.175 (0.875,1.579)	0.283	0.890 (0.672,1.179)	0.418	0.959 (0.804,1.143)	0.639	1.082 (0.859,1.362)	0.504
	Low HDL level	1.400 (1.151,1.701)	0.001	1.265 (0.979,1.635)	0.073	0.955 (0.710,1.285)	0.761	1.050 (0.804,1.373)	0.719	1.305 (1.113,1.529)	0.001	1.297 (1.045,1.610)	0.018
	High glucose	1.187 (0.981,1.436)	0.077	1.075 (0.843,1.370)	0.562	0.879 (0.663,1.165)	0.371	1.163 (0.915,1.478)	0.216	1.111 (0.953,1.296)	0.180	1.330 (1.083,1.633)	0.007
Model 3	No MetS	Ref.	Ref.	Ref.	Ref.	Ref.	Ref.	Ref.	Ref.	Ref.	Ref.	Ref.	Ref.
	High blood pressure	1.318 (1.084,1.601)	0.006	1.007 (0.782,1.296)	0.960	0.927 (0.694,1.237)	0.604	1.191 (0.930,1.527)	0.167	1.309 (1.120,1.531)	0.001	1.289 (1.289,1.041)	0.020
	^*^Abdominal obesity	1.085 (0.891,1.322)	0.417	1.271 (0.991,1.630)	0.059	1.169 (0.877,1.557)	0.287	1.012 (0.793,1.292)	0.925	1.079 (0.921,1.263)	0.345	1.087 (0.875,1.350)	0.453
	High triglycerides	1.073 (0.868,1.326)	0.516	0.907 (0.683,1.203)	0.497	1.166 (0.868,1.567)	0.308	0.881 (0.665,1.168)	0.379	0.952 (0.799,1.136)	0.587	1.072 (0.851,1.351)	0.555
	Low HDL level	1.393 (1.146,1.694)	0.001	1.271 (0.982,1.644)	0.068	0.949 (0.705,1.276)	0.728	1.034 (0.790,1.352)	0.809	1.301 (1.109,1.525)	0.001	1.284 (1.035,1.594)	0.023
	High glucose	1.202 (0.988,1.463)	0.066	1.089 (0.847,1.399)	0.507	0.861 (0.643,1.151)	0.313	1.108 (0.864,1.421)	0.418	1.121 (0.956,1.314)	0.159	1.303 (1.054,1.612)	0.015

Model 1 = unadjusted.

Model 2 = Model 1 + age, gender, and education level.

Model 3 = Model 2 + (smoking, coronary artery disease, hypertension, type 2 DM).

^*^Abdominal obesity is characterized by waist circumference greater than the cut-off value described in the context.

Abbreviations: HR: Hazard ratio; HDL: High-density lipoprotein cholesterol; MetS: Metabolic syndrome.

### Hazard ratios for dementia in different MetS-transitioning groups

A feature of our study is the classification of participants into different transitioning groups according to their metabolic status at both baseline and follow-up. We hypothesized that transitioning toward MetS would result in deteriorated cognitive function and vice versa. In Table 4, participants who transitioned to MetS and who maintained their MetS status had higher HR for dementia (normal-to-MetS HR: 1.243 [95% CI = 0.986–1.569, *p* = 0.066]; MetS-to-MetS HR: 1.362 [95% CI = 1.113–1.667, *p* = 0.003]) compared to the normal group. Interestingly, the HR for dementia of the MetS-to-normal group was 1.374 (95% CI = 1.054–1.792, *p* = 0.019) compared to the normal group, suggesting that reversing metabolic parameters did not bring about concurrent improvement of cognitive performance. Similar trends were observed in various cognitive domains (Table 5). For the domains revealing statistical significance (orientation, language, and visuospatial abilities), the HRs of MetS-to-normal group were all greater than 1, indicating an increased risk in this group. Importantly, the MetS-to-normal group showed higher HR than the normal-to-MetS group, suggesting that having MetS at baseline may serve as an unfavorable prognostic factor.

**Table 4:** Comparison of hazard ratios for dementia in different transitioning groups at baseline (year 2008) and follow-up (year 2018).

	**Variables**	**Dementia**	
		**HR (95% CI)**	***p* value**
Model 1	Normal → Normal	Ref.	Ref.
	Normal → MetS	1.243 (0.986,1.569)	0.066
	MetS → Normal	1.374 (1.054,1.792)	0.019
	MetS → MetS	1.362 (1.113,1.667)	0.003
Model 2	Normal → Normal	Ref.	Ref.
	Normal → MetS	1.195 (0.950,1.512)	0.127
	MetS → Normal	1.357 (1.040,1.769)	0.024
	MetS→MetS	1.319 (1.077,1.615)	0.007
Model 3	Normal → Normal	Ref.	Ref.
	Normal → MetS	1.207 (0.955,1.525)	0.116
	MetS → Normal	1.361 (1.043,1.777)	0.023
	MetS → MetS	1.363 (1.110,1.674)	0.003

Model 1 = unadjusted.

Model 2 = Model 1 + age, gender, and education level.

Model 3 = Model 2 + (smoking, coronary artery disease, hypertension, type 2 DM).

Abbreviation: HR: Hazard ratio.

**Table 5:** Comparison of change in hazard ratios of decline in different cognitive domains for patients with or without metabolic syndrome at baseline (year 2008) and follow-up (year 2018).

**Variables**		**Orientation**		**Registration**		**Concentration**		**Memory recall**		**Language**		**Visuospatial abilities**	
		**HR (95% CI)**	***p* value**	**HR (95% CI)**	***p* value**	**HR (95% CI)**	***p* value**	**HR (95% CI)**	***p* value**	**HR (95% CI)**	***p* value**	**HR (95% CI)**	***p* value**
Model 1	Normal→Normal	Ref.	Ref.	Ref.	Ref.	Ref.	Ref.	Ref.	Ref.	Ref.	Ref.	Ref.	Ref.
	Normal→MetS	1.015 (0.756,1.362)	0.921	1.174 (0.834,1.174)	0.359	0.873 (0.574,1.330)	0.528	0.907 (0.636,1.294)	0.590	1.046 (0.834,1.312)	0.695	1.353 (1.012,1.809)	0.041
	MetS→Normal	1.412 (1.037,1.922)	0.028	1.261 (0.847,1.875)	0.253	1.298 (0.853,1.977)	0.224	0.930 (0.607,1.426)	0.741	1.422 (1.123,1.801)	0.003	1.477 (1.057,2.063)	0.022
	MetS→MetS	1.664 (1.334,2.077)	<0.001	1.208 (0.891,1.637)	0.224	0.937 (0.653,1.343)	0.722	0.891 (0.645,1.229)	0.480	1.166 (0.961,1.416)	0.119	1.517 (1.178,1.952)	0.001
Model 2	Normal→Normal	Ref.	Ref.	Ref.	Ref.	Ref.	Ref.	Ref.	Ref.	Ref.	Ref.	Ref.	Ref.
	Normal→MetS	0.874 (0.650,1.175)	0.372	1.165 (0.827,1.642)	0.382	0.713 (0.467,1.088)	0.117	0.880 (0.616,1.257)	0.481	0.939 (0.747,1.179)	0.585	1.120 (0.835,1.501)	0.450
	MetS→Normal	1.280 (0.939,1.743)	0.118	1.181 (0.793,1.758)	0.413	1.136 (0.745,1.732)	0.553	0.844 (0.551,1.295)	0.438	1.316 (1.039,1.668)	0.023	1.319 (0.943,1.845)	0.105
	MetS→MetS	1.480 (1.185,1.850)	0.001	1.196 (0.882,1.623)	0.250	0.787 (0.548,1.130)	0.194	0.860 (0.623,1.188)	0.361	1.069 (0.880,1.299)	0.500	1.311 (1.017,1.689)	0.037
Model 3	Normal→Normal	Ref.	Ref.	Ref.	Ref.	Ref.	Ref.	Ref.	Ref.	Ref.	Ref.	Ref.	Ref.
	Normal→MetS	0.872 (0.647,1.174)	0.365	1.174 (0.831,1.659)	0.364	0.699 (0.456,1.070)	0.099	0.845 (0.589,1.210)	0.357	0.938 (0.746,1.180)	0.026	1.099 (0.818,1.476)	0.531
	MetS→Normal	1.273 (0.934,1.737)	0.127	1.190 (0.870,1.615)	0.394	1.122 (0.735,1.712)	0.594	0.819 (0.534,1.258)	0.362	1.311 (1.034,1.663)	0.026	1.295 (0.925,1.813)	0.132
	MetS→MetS	1.469 (1.171,1.843)	0.001	1.185 (0.798,1.774)	0.282	0.760 (0.526,1.097)	0.142	0.827 (0.596,1.148)	0.256	1.075 (0.882,1.309)	0.476	1.299 (1.003,1.682)	0.047

Model 1= unadjusted.

Model 2= Model 1 + age, gender, and education level.

Model 3= Model 2 + (smoking, coronary artery disease, hypertension, type 2 DM).

Abbreviation: HR: Hazard ratio.

### The percentage of cognitive decline in participants with MetS at baseline

We further investigated whether those with MetS at baseline were more likely to suffer from cognitive decline. Table 6 shows the percentage of each transitioning groups. The majority of participants with MCI at baseline either maintained their status (39.2%) or deteriorated to dementia (14.3%), whereas fewer showed improvement at follow-up (46.5%). Of participants who had dementia at baseline, 45.7% maintained their status, which were more than the other two improving subgroups. The above evidence revealed that having MetS at baseline may increase the risk of cognitive decline.

**Table 6:** Percentage of participants with metabolic syndrome at baseline in different cognitive-transitioning groups.

**Transition state**	**MetS (baseline)**
Baseline: MCI	
MCI → normal	46.5%
MCI → MCI	39.2%
MCI → dementia	14.3%
Baseline: dementia	
Dementia → normal	27.2%
Dementia → MCI	27.2%
Dementia → dementia	45.7%

Abbreviation: MCI: mild cognitive impairment.

## DISCUSSION

The association between MetS and cognition has been a much-discussed topic. Our study delineated the association between specific MetS symptoms and various cognitive domains to better understand their interrelationship. Furthermore, we investigated the effects of transitioning from normal metabolic state to MetS (or from MetS to normal metabolic state) on cognitive function. Our findings suggest that participants with MetS at baseline had persistent high risk of cognitive decline even when they restored to normal metabolic status.

Previous findings regarding the effects of individual MetS components on cognition are heterogeneous. Studies have demonstrated a link between each of the five metabolic components and cognition separately. Interestingly, those viewing MetS as a whole only revealed the risk of cognitive impairment for MetS itself, but failed to determine all five components in the same study [14–17]. Our study confirmed that high blood pressure, abdominal obesity, and low HDL levels were significantly associated with MCI/dementia. To the best of our knowledge, this is the first study to find a link between multiple MetS components and cognitive function in one comprehensive study.

An unanticipated result of this study was that the reverse of metabolic state from MetS to normal did not bring a concurrent reversal of cognitive impairments. This is contrary to previous studies which demonstrated that treating metabolic risk factors lowered the risk of cognitive decline [18, 19]. Extending our follow-up period may see noticeable risk reduction, but our results suggest that damage to the nervous system caused by MetS may be difficult to repair. Underlying mechanisms including neuroinflammation, oxidative stress, and decreased vascular reactivity all take time to restore to healthy status [6]. Thus, early detection and intervention of MetS may minimize the risk of long-lasting cognitive decline. This is supported by the “metabolic memory theory” [20] and “legacy effect” [21], which describe the benefits of early intensive treatment of hyperglycemia for preventing micro- and macro-vascular complications in type I and II diabetic patients. Renowned studies including Diabetes Control and Complications Trial (DCCT) [22], UK Prospective Diabetes Study (UKPDS) [23], and Veterans Administration Diabetes Trial (VADT) [24] showed reduced rates of cardiovascular comorbidities in long-term follow-up. Stopping the accumulation of oxidative stress and advanced glycation end products (AGEs) before vascular damage prevented the development of cardiovascular comorbidities [25]. Similarly, it is possible that the inability to reverse cognitive decline even when MetS was reversed was due to lack of early intervention during the 10-year follow-up period (from 2008 to 2018), illustrating the importance of earlier and stricter control of metabolic indices.

The three metabolic indices revealing significant correlations with cognition, each has a unique mechanism that contributes to neural damage. Regarding high blood pressure, two large-scale studies, each enrolling over 2000 Chinese community-dwelling older adults, also revealed that the prevalence of MCI was higher in hypertensive patients (both assessed with MMSE scores) [26, 27]. Our study recapitulated their findings using similar sample population and same evaluations. In 2016, the American Heart Association released a statement highlighting that the deleterious effects of hypertension are primarily due to cerebral vascular injury [28]. Hypertension remodels vascular structure of large, medium, and small vessels that ultimately leads to hypoperfusion or ischemia in regions critical for cognitive function [29]. With respect to abdominal obesity, a Korean study manifested the risk of cognitive decline in patients with increased BMI plus abnormal waist circumference (WC), but not in those with normal WC [30]. Whereas other studies use general measures of obesity as predictors of cognitive decline [31], the present study specifically focused on the adverse impact brought by abdominal obesity. The underlying mechanism linking obesity and cognitive impairment may be low grade inflammation within adipose tissue that gradually spreads to the brain, injuring vital regions responsible for cognitive functions [10]. Abdominal obesity may more authentically reflect the inability of subcutaneous adipose tissue to act as a metabolic buffer to store extra fat, causing fat accumulation in visceral organs [32]. As for low HDL level, three studies targeting middle-aged (mean age = 55) [33], old (mean age = 85.8) [34], and very old adults (mean age > 95) [35] suggested the correlation between HDL and cognitive function. HDL is involved in the regulation of amyloid β protein metabolism in the brain [36], and low levels of Apo A-I and A-II (the major apolipoproteins of HDL) were observed in Alzheimer’s disease [37]. The alteration of HDL level therefore exerts influence on cognitive performance. Collectively, our study is consistent with the previous literature regarding MetS components and cognitive decline.

Studies discussing the cognitive domains and MetS are relatively few as compared with those discussing MetS components. Concerning orientation, two studies utilizing the short blessed test (SBT), a six-item instrument evaluating orientation, registration, and attention, demonstrated the association between MetS and cognitive decline [38, 39]. SBT evaluates one of the same domains, orientation, as MMSE, and therefore our result supports their findings. Regarding language, a project performed by Boston University illustrated MetS adversely affecting the accuracy of lexical retrieval and sentence processing [40]. In respect of visuospatial ability, the evidence of MetS is lacking, but one longitudinal cohort study manifested the association between DM and lower levels of visuospatial ability [41]. As we are the few taking in-depth investigation into the affected cognitive domains, we call for more research on this issue to provide clinicians with clearer directions and planning of treatments.

Several limitations merit discussion in this study. First, we utilized MMSE as our cognitive evaluation tool since it is the only one available in Taiwan biobank. Nonetheless, some cognitive domains such as executive function, abstract reasoning, and perceptual-motor are under-represented in MMSE [42]. Secondly, despite the large number of participants, the study population is restricted to Taiwanese older adults, which may not be generalizable to a larger population. Further research is warranted to enhance the understanding in more cognitive domains and population of different ages and races.

## CONCLUSIONS

Our study highlighted the relationship between MetS and cognitive decline. Notably, individual MetS components that revealed significant correlations with cognitive function were high blood pressure, abdominal obesity, and low HDL level, while cognitive factors that correlated with MetS state were orientation, language, and visuospatial abilities. Furthermore, the transition of MetS to normal status in our study did not lower the risk of cognitive decline as anticipated, explaining the tough task of repairing neural damage. Therefore, early diagnosis and intervention of MetS may attenuate or prevent cognitive decline.

## MATERIALS AND METHODS

### Study population and study design

Participants in the current study were selected from the Taiwan biobank, a population-based research consortium that has been recruiting adults aged 30–70 years since 2008 [43]. With its large sample size, the goal of the Taiwan biobank is to facilitate the analyses of specific genes or biomarkers, improve treatment therapies, and promote prevention strategies for a variety of disorders. Participants were all free of cancer at entry, and written informed consent was collected before participation, which included an interview, physical examination, and biospecimen collection for each participant. The interviews and anthropometric measurements were performed by trained researchers with standardized questionnaires and devices. Detailed information is available on its official website (https://taiwanview.twbiobank.org.tw/index). This study was approved by the Institutional Review Board of Tri-Service General Hospital, Taipei, Taiwan.

This study enrolled a total of 5693 participants in 2008, and follow-ups were performed in 2018 (a ten-year follow-up). We included participants (*N* = 8630) with sufficient information about metabolic indices (measurement of blood pressure, waist circumference, serum lipid profile, and fasting glucose level) and completion of cognitive evaluation (MMSE), but further excluded those (*N* = 2937) who dropped off before follow-up (2018) during our study period. Participants were classified as either normal or as having MetS at both the beginning and end of the study, and cognitive evaluations were performed using the Mini-Mental State Examination (MMSE) at both the time points. Investigations into the detailed components of MetS and cognitive domains of MMSE were the highlights of our study.

### Definition of MetS

In the present study, we used a modified definition of MetS from the Third Report of the National Cholesterol Education Program’s Adult Treatment Panel [44], which was verified by the Health Promotion Administration of Taiwan [45]. The five criteria of MetS defined in this report are as follows: (1) abdominal obesity: waist circumference > 90 for men and > 80 cm for women; (2) hypertension: blood pressure ≥ 130/85 mmHg, or self-reported hypertension; (3) dyslipidemia: triglyceride (TG) ≥ 150 mg/dL (1.7 mmol/L); (4) dyslipidemia: high-density lipoprotein cholesterol (HDL-C) < 40 mg/dL (1.03 mmol/L) for men and < 50 mg/dL (1.3 mmol/L) for women; and (5) impaired glucose tolerance: fasting plasma glucose (FPG) ≥ 100 mg/dL, or a past history of diabetes status, or the use of antidiabetic agents. Participants meeting at least three of the above criteria were diagnosed as having MetS.

### Definitions of MCI and dementia

Participants were subjected to the MMSE for evaluating cognitive function. The MMSE consists of 11 questions and is a 30-point evaluation instrument that measures six cognitive domains: orientation, registration, attention and calculation, memory recall, language, and visuospatial abilities [46]. Mild cognitive impairment (MCI) was defined as a score of < 27 out of 30 for all subjects according to a study which validated an optimal cutoff value of Chinese version of MMSE in Taiwanese [47]. Dementia was defined as a score of < 17 for illiterate subjects, < 20 for subjects receiving only elementary education (less than 6 years), and < 24 for subjects receiving higher education. The above cutoff values have been verified for efficacy in several large studies in Chinese populations [48, 49].

### Covariates

Anthropometric measurements were performed by trained examiners. Body Mass Index (BMI) was calculated by dividing weight (in kilograms) by the square of height (in meters). Waist circumference was measured at the superior border of the iliac crest with a tape twice, and the average value was obtained for evaluation. Blood pressure was measured in a sitting position with the arm of the participants placed at the level of the right atrium. The right arm is measured unless there are contraindications. The mean of 3 readings of systolic and diastolic blood pressure was taken. Participants were told to fast at least 8 hours before taking blood tests. FPG and serum lipid profile levels (TG and HDL-C) were detected by a glucose oxidase method and an enzymatic colorimetric method respectively. Personal history including coronary artery disease, dyslipidemia, hypertension, DM, and smoking was obtained by a questionnaire administered by trained interviewers.

### Statistical analyses

Statistical analyses were performed using SPSS (IBM SPSS Statistics for Windows, Version 22.0; IBM Corp., Armonk, NY, USA). Analysis of variance and Pearson’s χ2 test were used to examine the differences of continuous and categorical variables, respectively. The Cox proportional hazards model was used to measure the effect of covariates on events of interest (for instance, the occurrence of MCI and dementia in our study) within a period of time. Kaplan–Meier curves were plotted to estimate the probability of an event at a respective time interval. Three extended models were provided for covariate adjustment: Model 1 = unadjusted; Model 2 = adjusted for age, gender, and education level; Model 3 = adjusted for age, gender, education level, smoking, coronary artery disease, hypertension and type 2 DM. *p* value of < 0.05 was considered statistically significant.

### Ethics approval and consent to participate

Taiwan biobank (TWB) is a publicly available data set and all participants in TWB provide written informed consent, consistent with approval from the Ministry of Health and Welfare of Taiwan. In addition, the ethical, legal, and social implications of this biobank abide by the specific regulation “Human Biobank Management Act" of Taiwan in order to ensure the rights and benefits of biological database participants.

### Data availability

Some or all data generated or analyzed during this study are included in this published article or in the data repositories listed in References. The datasets generated and analysed during the current study are available from the Taiwan biobank website, (https://taiwanview.twbiobank.org.tw/index).

## Supplementary Materials

Supplementary Table 1
