# Supplementary material for: Longitudinal trajectories of metabolic syndrome on different neurocognitive domains: a cohort study from the Taiwan biobank
Source: Aging (Albany NY). 2021 Jun 11;13(11):15400–12. doi: 10.18632/aging.203099 (PMC8221342; doi:10.18632/aging.203099)
Supplement: Supplementary Table 1 [file aging-13-203099-s001.pdf]

SUPPLEMENTARY TABLE

Supplementary Table 1. Hazard ratios of the number and content of metabolic syndrome components for elderly participants with MCI and dementia.

MCI						
**Variables**	**Model 1**		**Model 2**		**Model 3**	
	**HR (95% CI)**	***p* value**	**HR (95% CI)**	***p* value**	**HR (95% CI)**	***p* value**
**No metabolic syndrome**	**Ref.**	**Ref.**	**Ref.**	**Ref.**	**Ref.**	**Ref.**
Number of metabolic syndrome components						
1	1.193 (1.035,1.377)	0.015	1.181 (1.024,1.362)	0.023	1.177 (1.020,1.358)	0.026
2	1.282 (1.110,1.480)	0.001	1.217 (1.053,1.406)	0.008	1.207 (1.044,1.395)	0.011
3	1.244 (1.061,1.458)	0.007	1.199 (1.023,1.046)	0.025	1.179 (1.004,1.385)	0.045
4,5	1.503 (1.263,1.788)	<0.001	1.421 (1.194,1.692)	<0.001	1.402 (1.175,1.672)	<0.001
p for trend	<0.001		0.002		0.002	
Components of metabolic syndrome						
High blood pressure	1.203 (1.093,1.323)	<0.001	1.193 (1.085,1.313)	<0.001	1.193 (1.083,1.315)	<0.001
*Abdominal obesity	1.137 (1.038,1.247)	0.006	1.073 (0.976,1.180)	0.144	1.065 (0.968,1.172)	0.197
High triglycerides	1.028 (0.923,1.145)	0.613	1.014 (0.910,1.129)	0.800	1.007 (0.904,1.122)	0.901
Low HDL level	1.149 (1.038,1.271)	0.007	1.125 (1.016,1.245)	0.024	1.116 (1.007,1.236)	0.036
High glucose	1.092 (0.994,1.200)	0.068	1.088 (0.989,1.197)	0.083	1.070 (0.969,1.180)	0.181
**Dementia**						
**Variables**	**Model 1**		**Model 2**		**Model 3**	
	**HR (95% CI)**	***p* value**	**HR (95% CI)**	***p* value**	**HR (95% CI)**	***p* value**
**No metabolic syndrome**	**Ref.**	**Ref.**	**Ref.**	**Ref.**	**Ref.**	**Ref.**
Number of metabolic syndrome components						
1	1.698 (1.277,2.259)	<0.001	1.531 (1.151,2.037)	0.003	1.539 (1.157,2.049)	0.003
2	1.946 (1.464,2.587)	<0.001	1.566 (1.176,2.087)	0.002	1.575 (1.181,2.100)	0.002
3	1.956 (1.443,2.650)	<0.001	1.559 (1.149,2.117)	0.004	1.568 (1.153,2.133)	0.004
4,5	2.298 (1.657,3.187)	<0.001	1.800 (1.296,2.499)	<0.001	1.814 (1.303,2.527)	<0.001
p for trend	<0.001		0.001		0.002	
Components of metabolic syndrome						
High blood pressure	1.345 (1.141,1.585)	<0.001	1.282 (1.087,1.512)	0.003	1.308 (1.106,1.548)	0.002
Abdominal obesity	1.442 (1.223,1.700)	<0.001	1.149 (0.967,1.365)	0.115	1.147 (0.965,1.364)	0.120
High triglycerides	1.213 (1.014,1.451)	0.035	1.137 (0.950,1.361)	0.161	1.132 (0.946,1.356)	0.176
Low HDL level	1.364 (1.150,1.617)	<0.001	1.247 (1.050,1.480)	0.012	1.241 (1.045,1.474)	0.014
High glucose	1.069 (0.906,1.262)	0.427	1.037 (0.878,1.224)	0.670	1.027 (0.865,1.220)	0.761

Model 1 = unadjusted.
Model 2 = Model 1 + age, gender, and education level.
Model 3 = Model 2 + (smoking, coronary artery disease, hypertension, type 2 DM).
*Abdominal obesity is characterized by waist circumference greater than the cut-off value described in the context.
Abbreviations: HR: Hazard ratio; HDL: High-density lipoprotein cholesterol.
